# Lactadherin orthologs inhibit migration of human, porcine and murine intestinal epithelial cells

**DOI:** 10.1002/fsn3.479

**Published:** 2017-05-15

**Authors:** Steffen Nyegaard, Trine Andreasen, Jan Trige Rasmussen

**Affiliations:** ^1^ Department of Molecular Biology University of Aarhus Aarhus C Denmark

**Keywords:** IBD, intestinal cell migration, lactadherin, MFG‐E8, wound healing

## Abstract

Lactadherin was originally described due to its appearance in milk, but is abundantly expressed especially by professional and nonprofessional phagocytes. The proteins has been shown to have a multitude of bioactive effects, including inhibition of inflammatory phospholipases, induction of effero‐ and phagocytosis, prevent rotavirus induced gastroenteritis, and modulate intestinal homeostasis by regulating epithelial cell migration. The level of expression seems to be important in a row of serious pathologies linked to the intestinal epithelial barrier function, vascular‐ and autoimmune disease. This study examines the ability of lactadherin to modulate migration of intestinal epithelium. A cell exclusion assay is used to quantify the ability of human, bovine and murine lactadherin orthologs to affect migration of primary small intestine epithelium cells. Previous reports show that recombinant murine lactadherin stimulate rat small intestine cell migration. The present study could not confirm this. Conversely, 10 μg/ml lactadherin inhibits migration. Therefore, as lactadherins enteroprotective properties is well established using in vivo models we conclude that the protective effects are linked to lactadherins ability operate as an opsonin, or other modulating effects, and not a direct lactadherin‐cell induction of migration. Thus, the molecular mechanism behind the enteroprotective role of lactadherin remains to be established.

## 1 INTRODUCTION

Lactadherin, also called milk fat globule epidermal growth factor 8 (MFG‐E8), is a 409 amino acid glycoprotein which has been increasingly investigated and found to play multiple roles in diverse cellular interactions important in both normal biology and states of disease, for example, macrophage phagocytosis, inflammation, adult onset‐autoimmune lupus‐like pathology, splenomegaly, and defective germinal center formation (for review see (Raymond, Ensslin, & Shur, 2009; Aziz, Jacob, Matsuda, & Wang, 2011)). Bovine lactadherin comprises two N‐terminal epidermal growth factor homology domains and the C‐terminal region consists of two C domains (C1 and C2), which share homology with the lipid‐binding “C” domains of blood coagulation factor VIII and factor V. These C domains, and especially the C2 domain, confer ability to bind phosphatidylserine (PS) specifically in both the coagulations factors and lactadherin. The second epidermal growth factor (EGF) domain contains an Arg‐Gly‐Asp (RGD) motif that is able to interact with extracellular receptors, namely the integrins α_v_β_5_ and α_v_β_3_ (Andersen, Berglund, Rasmussen, & Petersen, 1997). It was hypothesized that lactadherin by the two‐sided binding affinities could operate as an opsonin facilitating PS‐dependent phagocytosis of apoptotic cells (Andersen, Graversen, Fedosov, & Petersen, 2000) which was consecutively confirmed in vivo (Hanayama, Tanaka, Miwa, & Shinohara, 2002). Successively, numerous studies used lactadherin or lactadherin domains to detect and/or measure the exofacial phosphatidylserine and intracellular PS pools (see (Shi, Shi, Waehrens, & Rasmussen, 2006; Yeung, Gilbert, Shi, & Silvius, 2008; Waehrens, Heegaard, Gilbert, & Rasmussen, 2009; Fairn, Schieber, Ariotti, & Murphy, 2011) for examples).

The phospholipid‐binding characteristics of lactadherin was investigated thoroughly by stopped flow kinetics (Otzen, Blans, Wang, & Gilbert, 2012). The kinetic data suggests a two‐step binding mechanism, including an initial binding followed by a slower step that might reflect either a conformational change or a different way of membrane insertion. It was possible to detect binding to not more than 0.03% PS and higher concentrations of PS increased the association kinetics and the affinity (Otzen et al., 2012).

Consumption of feed containing lactadherin seems to protect against infection with rotavirus (Kvistgaard, Pallesen, Arias, & López, 2004; Newburg, Ruiz‐Palacios, & Morrow, 2005), and the protein has also been shown to hinder attachment of enterotoxic *E. coli* to porcine small intestinal villi (Shahriar, Ngeleka, Gordon, & Simko, 2006). Furthermore, it was suggested that lactadherin has the ability to influence intestinal maintenance and repair in inflammatory bowel models by inducing the migration rate of rat small intestine cells (Bu, Zuo, Wang, & Ensslin, 2007). This effect was efficacious from as little as 2 nmol/L recombinant murine lactadherin (long isoform) and a three‐fold increase in migration was observed at 10 n mol/L. Using siRNA and a selective inhibitor against PKCε, Bu et al. (2007) showed that the lactadherin induced migration could be abolished. Bu et al. (2007) further showed that lactadherin depletion in mice by hamster anti‐murine MFG‐E8 antibody entailed mild focal mucosal injury at villous tips and an increase in necrotic epithelial cells as well as decreased epithelial migration. Cecal ligation and puncture was used to introduce sepsis derived intestinal damage in wild type and lactadherin knockout mice. Treatment of the septic mice with 2 mg/kg lactadherin, *i.p*. decreased the pathological phenotype and increased epithelial migration. Accordingly, lactadherin knockout mice displayed worse disease phenotype than wild type mice. In similar mice studies by Aziz, Ishihara, Mishima, and Oshima (2009) a beneficial effect of lactadherin was seen on survival rate, intestinal length and inflammatory markers (IL‐1β, TNF‐α). These promising experiments using murine cell and animal models incited us to investigate the ability of lactadherin to modulate enterocyte migration rate across different lactadherin orthologs and enterocyte species. Purified human, bovine and recombinant long isoform murine lactadherin are tested, covering phylogenetic variation in primary structure as well as differing post translational modification patterns. To comprehensively determine the migratory response of epithelia from distinct intestinal sections in commonly used cells, we utilized nontumorigenic pig jejunum cells (IPEC‐J2) (Schierack, Nordhoff, Pollmann, & Weyrauch, 2006), nontumorigenic rat ileum cells (IEC‐18) (Quaroni, Wands, Trelstad, & Isselbacher, 1979), nontumorigenic human fetal small intestine cells (FHs‐74 int) (Owens, Smith, Nelson‐Rees, & Springer, 1976) and human colonic carcinoma cells (Caco‐2) (Fogh, Wright, & Loveless, 1977). In this study an optimized migration assay was used to distinguish between migration and inflammation derived chemotaxia. Furthermore, basal membrane extract was utilized for optimal biological relevance and to avoid adverse effects of sample protein adsorption. In contradiction with previous reports, none of the lactadherin orthologs induced statistically significant migration on any of the enterocyte cell lines tested.

## 2 MATERIALS AND METHODS

### 2.1 Materials

The intestinal epithelial cells used were IEC‐18 (rat, ATCC# CRL‐1589), FHs‐74 int (human, ATCC# CCL‐241), Caco‐2 (human, DSMZ**#** ACC 169) and IPEC‐J2 (pig) were obtained from LGC Standards AB (Boras, Sweden), Deutsche Sammlung von Mikroorganismen und Zellkulturen (Braunschweig, Germany) and as a kind gift from assoc. prof. Stine Brandt Bering, Copenhagen University, Denmark. Dulbecco's modified Eagle medium (DMEM), fetal bovine serum (FBS), penicillin/streptomycin, recombinant EGF, SYTO‐24^®^ DNA stain (cat# S7559) and CellMask Orange (cat# C10045) were purchased at Life Technologies, Denmark. Recombinant human insulin was purchased from Sigma‐Aldrich, Denmark. Basal membrane extract (BME) (growth factor reduced Cultrex^™^) and recombinant murine long isoform lactadherin (rmLact, cat# 2805‐MF‐050) was purchased at R&D systems, Denmark. The Oris migration system was used as donor for our optimized migration assay (Platypus technologies, WI). Clear bottom, 96‐well plates were from Nunc, Denmark (cat# 165305). All chemicals and solutions were analytical grade and endotoxin free when applicable. Images were acquired using Olympus Cell^B version 3.3 (build 2108) software. Image processing and nuclei counting was done in ImageJ v1.43u using maximum resolution (4140x3096pixel) TIFF files. Microsoft Excel 2010 and Graphpad Prism v5 was used for data handling and statistical analysis.

### 2.2 Purification of bovine and human lactadherin

Bovine lactadherin (bLact) was purified from fresh milk essentially as described by Hvarregaard, Andersen, Berglund, and Rasmussen (1996). Analysis by SDS‐PAGE and N‐terminal amino acid sequencing showed that the used material had a purity above 97% and contained both glycosylation variants in comparable amounts. Human lactadherin (hLact) with a purity >95% was purified as described by Kvistgaard et al. (2004).

### 2.3 Maintaining intestinal cell lines

IEC‐18, Caco‐2 and IPEC‐J2 were maintained in DMEM + 10% v/v FBS + 1% v/v penicillin/streptomycin and subcultured at 70%–80% confluence (1:7–1:10 three times a week). Caco‐2 cells were between passage 5 to 25 during the experiment and IEC‐18 and IPEC‐J2 cells between three and 15. FHs‐74 int cells were cultured using DMEM + 10% v/v FBS + 1% v/v penicillin/streptomycin +10 ng/ml recombinant human insulin at 37°C, 5% CO_2_ and subcultured three times a week at 1:20. The FHs‐74 int cells were monitored for morphological changes and experiments carried out between passage three and 15. The starvation media FBS concentration in the assay was found by titrating FBS concentration versus cellular morphology changes, cell death and mitosis as described in Nyegaard, Christensen, and Rasmussen (2016).

### 2.4 Generating consistent wounds

Reproducible wounds were generated by utilizing a modified version of the Platypus Oris assay (Nyegaard et al., 2016). The silicone inserts were rinsed in 70% ethanol, washed in growth media and mounted in a BME coated, clear bottom, 96‐well plate. Experiments were done with BME coating to negate any possible haptotaxic effects of surface coating the wells with milk proteins as well as to provide a physiological relevant substrate. The outmost rows and columns were avoided due to rim effects, but with added media to buffer the temperature of the adjacent wells. 100,000 cells were seeded per well around the inserts to reach almost immediate confluence. After 24 hr incubation the insert was removed and bioactive components dissolved in DMEM + 1% FBS v/v + 1% v/v pen/strep were added. Upon another 24 hr incubation the plate was washed in 37°C DMEM w/o phenol red, stained with appropriate fluorophore, washed twice and immediately transferred to image acquisition.

In experiments using lactadherin coated polystyrene, bLact was dissolved in PBS without MgCl_2_ or CaCl_2_ buffer, 100 μl coating solution added to each well and incubated overnight at 4°C. The plates were subsequently washed twice in 300 μl PBS without MgCl_2_ or CaCl_2_ buffer immediately before use.

### 2.5 Staining nuclei and plasma membrane for quantification of migration and morphological studies

Visualization of cell nuclei was done by addition of SYTO‐24^®^ DNA stain to a total concentration of 2 μmol/L followed by 30 minutes incubation. Plasma membrane visualization was done using a final concentration of 5 μg/ml CellMask orange and 5 minute incubation. Excess stain was removed by gentle wash using 2x200 μl 37°C DMEM with 25 mmol/L HEPES and no phenol red.

### 2.6 Image acquisition and data processing

Image acquisition was done on a Leica DMI 3000B coupled to an Olympus DP72 image sensor with a Leica H/PLAN 4X/0.10, a 10X/0.25 NPLAN and a L 20X/0.4 CORR HXPL objective. 470/40:525/50 excitation and emission filters were used for SYTO‐24^®^ fluorescence image acquisition and 546/12:605/75 excitation and emission filter for CellMask^®^ Orange acquisition. Differential interference contrast images were acquired using the appropriate Nomarski prism and polarizing filters combined with a 10x objective.

IEC‐18 experiments where performed as independent triplicates of six and all other experiments in sextuplicates. Image processing and nuclei counting was done in ImageJ v 1.43u with 30 μm^2^ minimum threshold. IPEC‐J2 and Caco‐2 cells both stained irregularly with SYTO‐24 so migration was quantified as closure of area instead of nuclei count by manually drawing masks.

### 2.7 Statistical analysis

Data processing was done in Microsoft Excel 2010 and Graphpad Prism v5 and statistical significance calculated using a two‐tailed Welch's *t*‐test. * denotes *p* < .05; *^*^ *p* < .01 and *^**^ *p* < .001.

## 3 RESULTS

**Figure 1:**
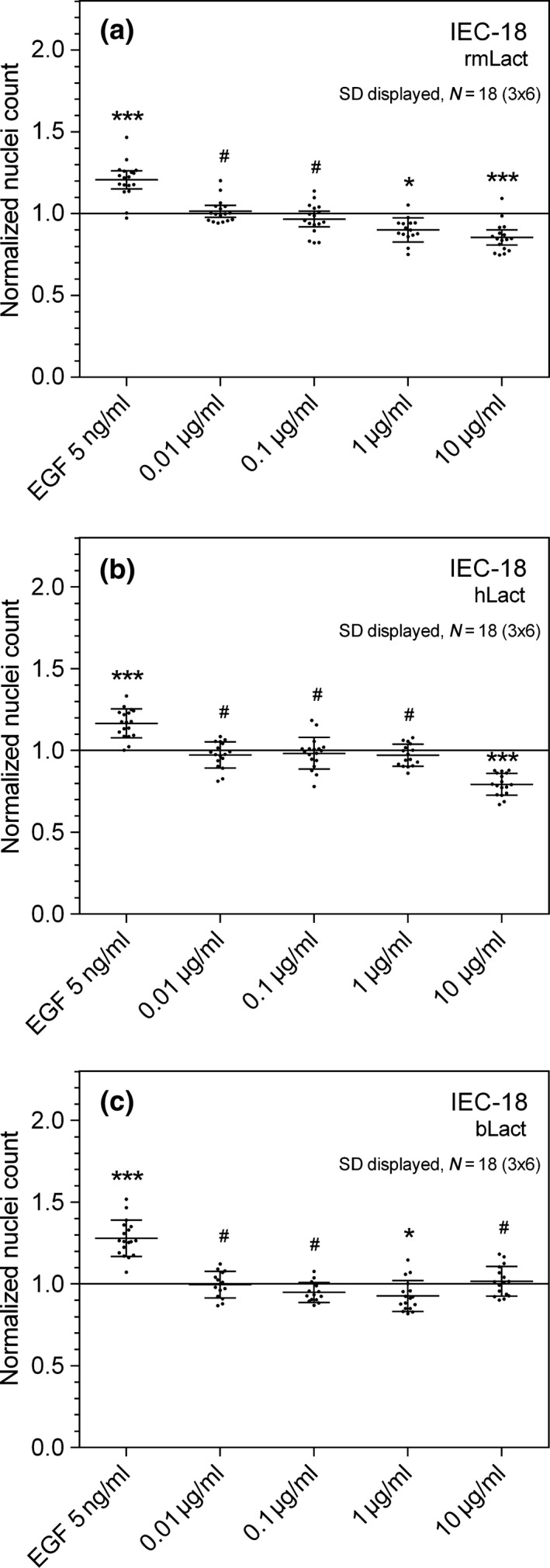
Rat small intestine cells (IEC‐18) were seeded in the cell exclusion assay, grown to confluence, the insert removed and the cells treated with a concentration gradient of lactadherin orthologs as per materials and methods. As seen, IEC‐18 migration is proportionally inhibited to a maximal 14.6% inhibition versus control monolayers using rmLact (a), 21% inhibition using hLact (b) and no statistically significant inhibition using bLact (c). * denotes *p* < .05, ***p < .001, and # not statistically significant

Initially IEC‐18 cells (rat) were grown and used in the optimized cell migration assay to comprehensively test the ability of lactadherin to modulate the IEC‐18 cell migration rate. To begin with effects of recombinant long isoform of murine lactadherin (rmLact) on cell migration was tested in a gradient spanning from 0.01 to 10 μg/ml (0.217 nmol/L to 0.217 μmol/L) and compared to experiments without protein supplements. Introduced cell free areas (“wounds”) in the IEC‐18 cell layer turned out to be gradually difficult to cover by migrating cells by increasing content of rmLact (Figure 1a). In this experiment that was repeated six times with independent triplicates a 14.6% inhibition of the cell migration rate was seen with presence of 10 μg rmLact/ml, whereas no statistically significant effects were observed using 0.01 μg/ml or 0.1 μg/ml of rmLact. EGF (5 ng/ml) was used as positive control resulting in a 20.7% increased migrational rate. Next, purified bovine and human lactadherin was added on IEC‐18 wounds using an equivalent set of protein concentrations to evaluate bioactive effects of these two naturally derived lactadherin orthologs. As seen in Figure 1b concentrations of 1 μg/ml and lower hLact had no measurable effect on the cell migration rate, whereas a 21% inhibition was observed when the concentration was raised to 10 μg/ml. Bovine lactadherin displayed little to no effect on IEC‐18 migration.

**Figure 2:**
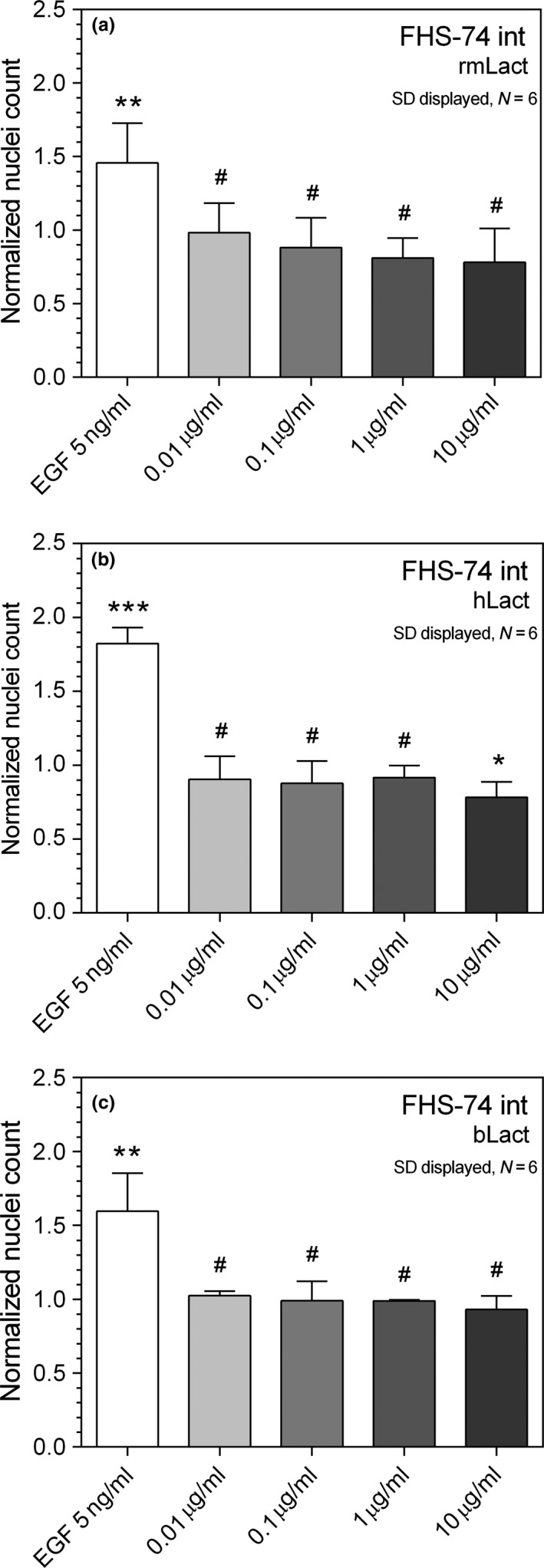
Human primary small intestine cells (FHS 74 int) were likewise grown in the cell exclusion assay and treated with a concentration gradient of lactadherin orthologs. Recombinant murine lactadherin exhibited an inhibitory trend, however was not statistically significant (a). Human lactadherin inhibited migration by 21.8% (b) and bLact did not affect migration (c). * denotes *p* < .05, **p < .01, ***p < .001, and # not statistically significant

All lactadherin orthologs were subsequently added to FHs‐74 int (human) monolayer wounds. As the results were tightly spaced with no significant change between six and 18 replicates, six were used using FHs‐74 int cells. Recombinant long isoform of murine lactadherin had a more pronounced effect on FHs‐74 int cell migration. Despite not being statistically significant due to slightly elevated standard deviation on the terminal data point, a proportional trend of inhibition versus rmLact concentration was observed with the highest inhibition at 10 μg/ml of 22% (Figure 2a). Human lactadherin could only elicit significant inhibition at 10 μg/ml and reduced migration to 21.8% (Figure 2b) and presence of bovine lactadherin did not have any significant inhibitory effects at concentrations up to 10 μg/ml (Figure 2c).

**Figure 3:**
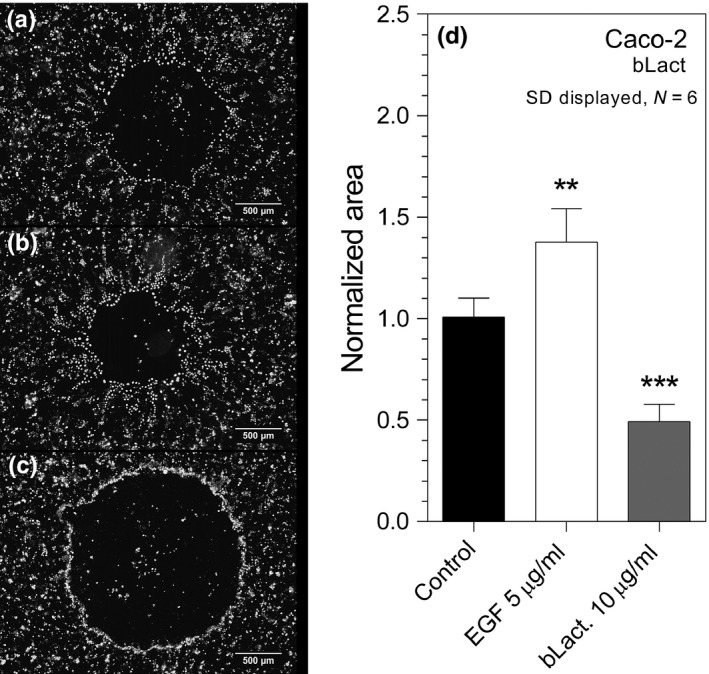
Human colonic carcinoma cells (Caco‐2) were likewise treated with 10 μg/ml bLact and micrographs acquired of the cell exclusion areas. A striking inhibition was observed between control wounds (a) and bLact treated wound (c). As a positive control wounds were treated with 5 ng/ml epidermal growth factor which induced migration as expected (b). Quantification of remaining void area post treatment was done on sextuplicates and showed a 51% reduction of migration when Cac0‐2 monolayers were treated with 10 μg/ml bLact (d). **denotes *p* < .01, and ***p < .001

**Figure 4:**
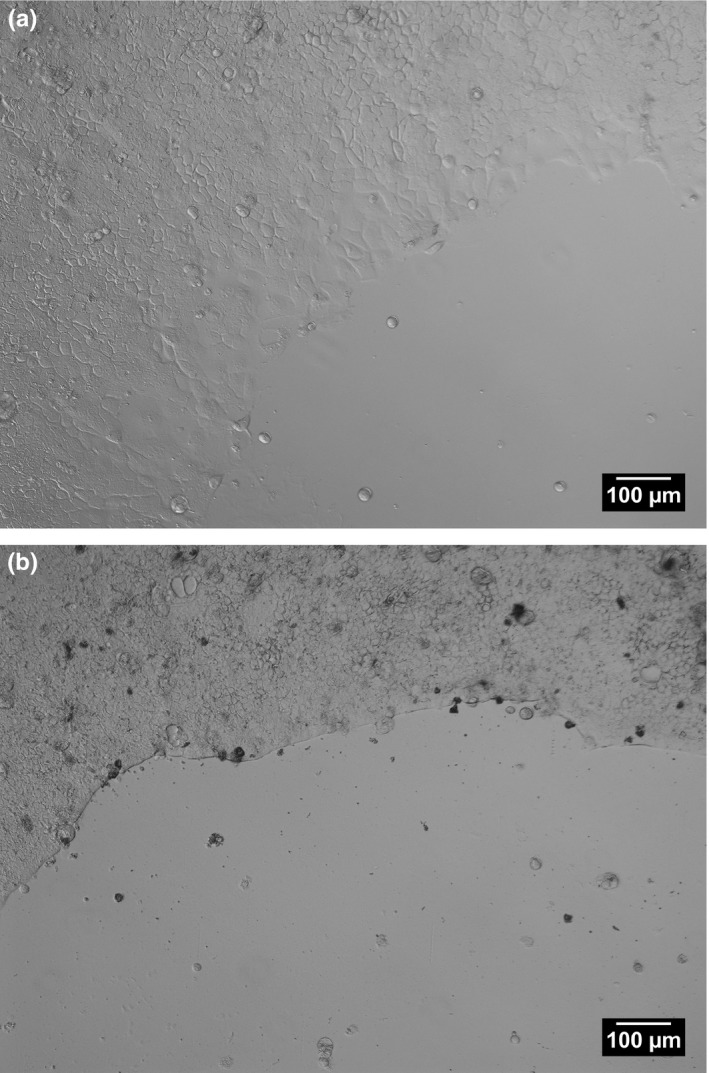
Caco‐2 monolayers were treated with 10 μg/ml bLact and examined using Differential Interference Contrast. Control monolayers (a) migrated collectively and displayed cobblestone morphology within the monolayer and a flattening of the pioneer cells with lamellapod extension in the axis of migration. Lactadherin‐inhibited pioneer cells (b) displayed no flattening or lamellapods and negligible wound closure was observed. This phenotype after treatment was similar to that of the pioneer cell in the IPEC‐J2 monolayers

**Figure 5:**
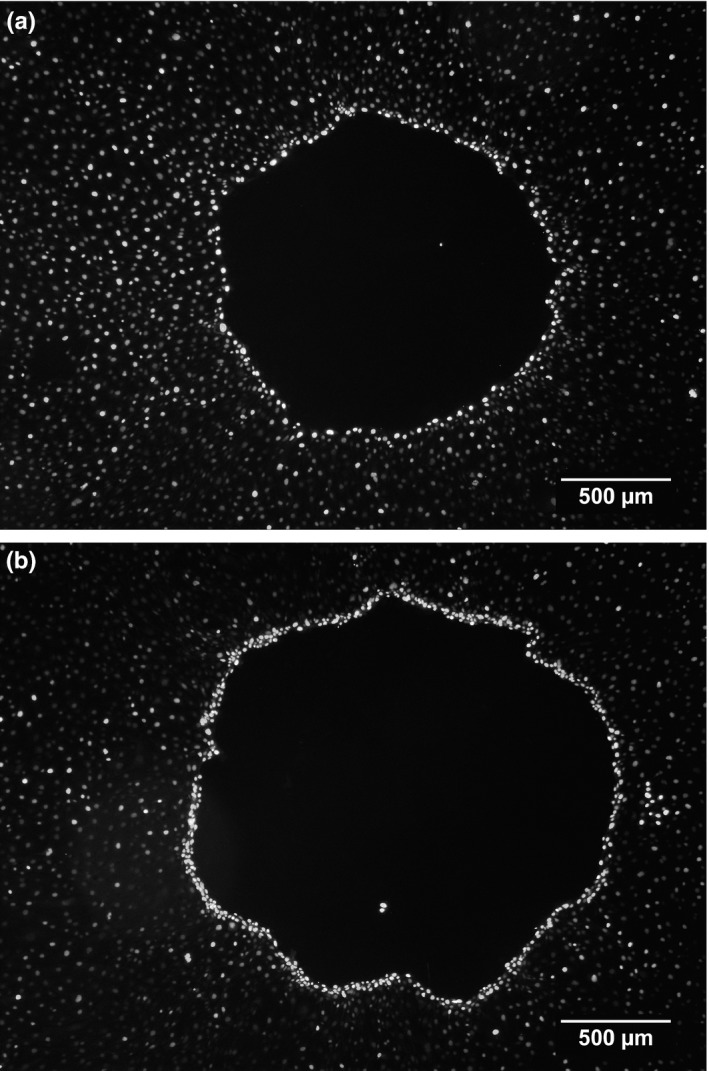
Primary pig small intestine cells (IPEC‐J2) were treated with 10 μg/ml bLact per materials and methods, the nuclei stained and micrographs acquired. When comparing control monolayers (a) with lactadherin‐treated monolayers (b) a pronounced ring of nuclei as well as a significantly larger exclusion area was observed in the latter

**Figure 6:**
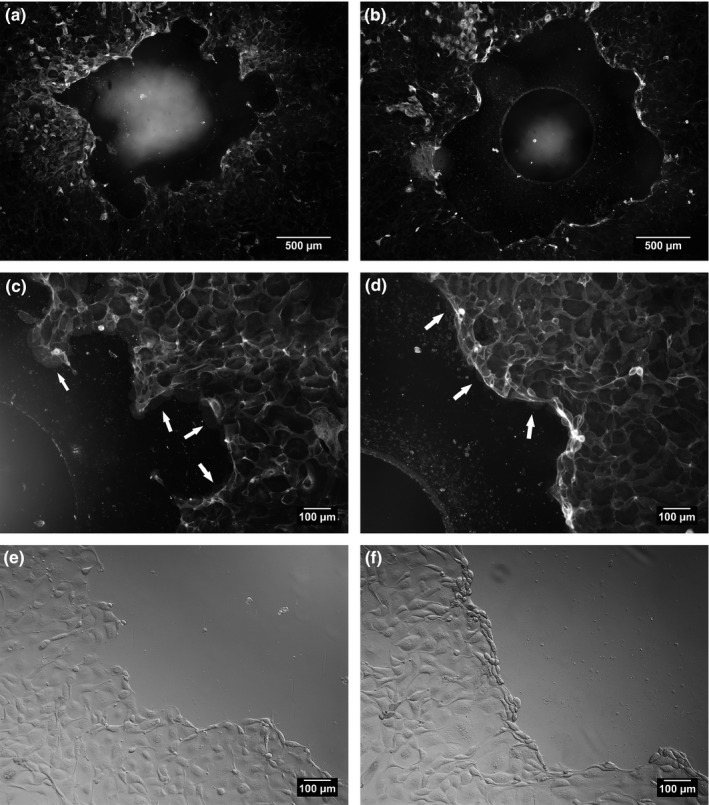
IPEC‐J2 cells treated with bLact and the plasma membrane visualized using Cellmask orange. Left panels (a, c & e) are without and right panels (b, d & f) are with 10 μg/ml bLact treatment. Whole wound comparison of control versus treated monolayers (a vs. b) exhibited the same wound morphology as described previously with less collective migration and wound coverage in bLact treated wells. Further magnification revealed a pronounced decrease in leading edge (arrows in c), but not cryptic lamellapod formation (arrows in c), and an accumulation of cells at the wound edge compared to control (d vs. c respectively). Examination of the leading edge using Differential Interference Contrast imaging revealed that the pioneer cells lost their flattened morphology characteristic of adequate adherence when treated with bLact. compared to controls (f vs. e)

To investigate the observed inhibitory effects of lactadherin on cell migration further, Caco‐2 (human) cells and IPEC‐J2 (pig) were incubated with a gradient of bLact however only 10 μg/ml exhibited statistically significant inhibition of migration and hence their morphology was studied using nuclear and plasma membrane stain as well as Differential Interference Contrast (DIC) microscopy (51% inhibition, Figure 3d). Cell‐free areas were introduced in Caco‐2 cell monolayers and marked reduction in cell migration was observed in the presence of 10 μg/ml bLact (Figure 3c), as opposed to experiments without additional protein supplementation (Figure 3a). 5 ng/ml EGF was used as positive migration stimulation controls (Figure 3b). Interestingly, bovine lactadherin treatment led to Caco‐2 cells accumulation near the wound edge (Figure 3c). The clear differences in the way Caco‐2 cells migrated during wound closure with and without lactadherin was readily observable by the use of DIC imaging (Figure 4). A marked pioneer cell border appears and little to no lamellipodia was seen in wounds treated with bovine lactadherin (Figure 4b). This is in contrast to the morphology seen in the controls (Figure 4a). This morphological change was similar to that of IPEC‐J2 cells treated with bLact. Addition of 10 μg/ml bLact to IPEC‐J2 cells inhibited wound closure by 35% in comparison to controls (Figure 5). Similar to Caco‐2 monolayers, a distinct fluorescent ridge was seen in the lactadherin treated wounds (Figure 5b), indicating that the cells accumulated at the wound edge. To investigate this event further, studies were made looking at cell morphology using plasma membrane stain and DIC imaging. Whole wounds treated with 10 μg/ml bovine lactadherin displayed marked differences in wound closure and overall morphology when compared to control. A clear polarization and a distinct leading edge of pioneer cells appeared when no lactadherin was added (Figure 6a). In contrast, it was seen that presence of lactadherin resulted in a dramatic reduction of protruding cells at the wound edge (Figure 6b). Further magnification clearly showed very few lamellipodia protrusions on leading edge pioneer cells upon lactadherin treatment (Figure 6d), whereas plentiful lamellipodia formations were seen in the controls (Figure 6c). It's of notice that the IPEC‐J2 cells migrated so that cryptic lamellipodia appeared (Figure 6c). A morphological feature, which is also seen by others applying the frequently used epithelia model with Madin‐Darby canine kidney (MDCK) cells (Farooqui & Fenteany, 2005; Fenteany, Janmey, & Stossel, 2000). Visualizing the wound edge using DIC confirmed that epithelial cells were in fact accumulating at the wound edge (Figure 6f) when compared to controls (Figure 6e).

**Figure 7:**
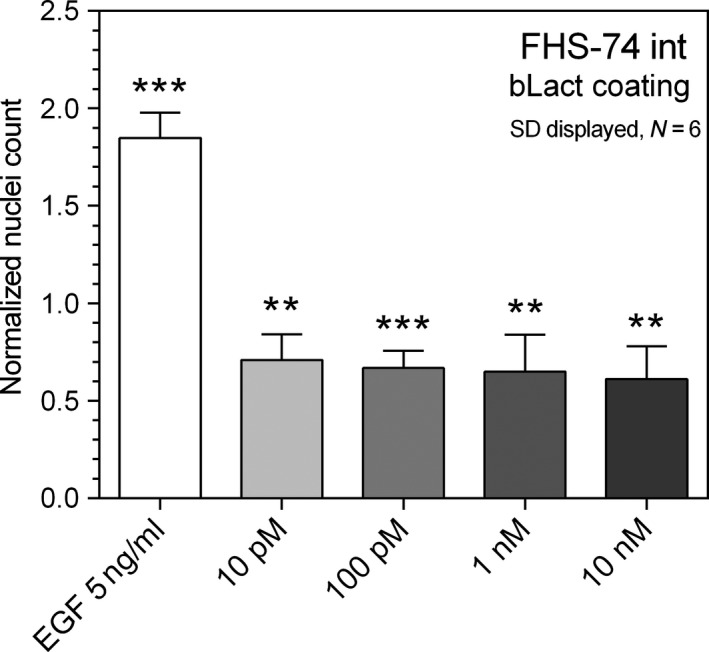
As lactadherin exhibits amphipathic properties and contains an integrin‐binding RGD domain it might induce migration by haptotaxia. To examine this, plates were coated with a 10 pmol/L–10 nmol/L bLact gradient and FHs‐74 int monolayers grown in the exclusion assay. All concentrations inhibited both cell adhesion when grown and cell migration into the voids. **denotes *p* < .01, and ***p < .001

As lactadherin displays amphipathic properties owed to its membrane binding discoidin domains and its hydrophilic and glycosylated N‐terminal tail, we investigated whether the inducing effect of lactadherin could relate to haptotaxic effects or due to general protein surface adsorption of lactadherin to the polystyrene tissue culture plates. To this end, wells were precoated with increasing amounts of lactadherin (10 pmol/L–10 nmol/L). As seen in Figure 7, precoating with lactadherin reduced the migratory rate of FHS‐74 int monolayers from 30 % (10 pmol/L) to 39 % (10 nmol/L). Furthermore, it's observed that monolayer morphology was disrupted, integrity lost and cells sparsely distributed (not shown).

## 4 DISCUSSION

The presented experiments were designed to study the ability of human, bovine and murine lactadherin protein orthologs to affect migration of intestinal cells. Three types of cultured cells and coating with basal membrane extract was used to cover a broad range of intestinal epithelial cell morphology, cell surface receptor expression, and physiological relevant set up. The tested protein samples were obtained by either recombinant expression (murine lactadherin) as well as purified lactadherin from human or bovine milk.

Prior to this work, it has been suggested that the long form of recombinant murine lactadherin (2–10 nmol/L) could promote the migration of rat IEC‐18 cells (Bu et al., 2007). In contrast to that, none of the presently investigated lactadherin orthologs displayed ability to enhance intestinal cell migration. Actually the opposite effect was observed, for exapmle, recombinant murine lactadherin reduced IEC‐18 and FHs‐74 int cell migration by 14.6% and 22% upon addition of 0.217 μmol/L protein and at lower, but more comparable range to Bu et al. (2007)no statistically significant effects were observed. In line with that, high amounts of human lactadherin decreased both IEC‐18 and FHs‐74 int cell migration with more than 20%. Interestingly, presence of 0.217 μmol/L bovine lactadherin did not affect IEC‐18 or FHs‐74 int cell mobility, whereas it reduced the migration of IPEC‐J2 and Caco‐2 cells. This discrepancy is likely to reflect individual differences between the types or species of cells, their differential expression of integrin subtypes or the effect of varying glycosylation and phosphorylation states of lactadherin from different sources. Microscopic inspection reveals that the phenotype and morphology of the FHs‐74 int and IEC‐18 cells is relatively similar. Unlike those the Caco‐2 cells display distinct columnar morphology with discernable tight and adherence junctions typical of colonic cells. The IPEC‐J2 cells is characterized by yet a distinct morphology with a two to three fold increase in diameter and pronounced cryptic lamellipodia, a phenotype also observed in MDCK cells (Farooqui & Fenteany, 2005). All monolayers displayed cell‐cell adherence, sheet migration with pioneer cells extending lamellapods as usually observed in migrating 2D monolayers (Farooqui & Fenteany, 2005; Vaughan & Trinkaus, 1966). In the case of Caco‐2 and IPEC‐J2 cells, the wound closure share similarities with the “purse string” model (Martin & Lewis, 1992) and the observed lactadherin‐induced inhibition of migration resembles that of Rac inhibited pioneer cells in MDCK monolayers (Fenteany et al., 2000). As Rho, Rac and Cdc42 GTPases play a pivotal role in the organization of the actin cytoskeleton, the Rac family mediates lamellapod actin polymerization and has been shown critical to cell spreading in conjunction with Cdc42 upon integrin activation (Price, Leng, Schwartz, & Bokoch, 1998; Tapon & Hall, 1997), further studies should be done to investigate the lack of GTPase activation or possibly inhibition as mediated by lactadherin.

Entero‐protective potential of lactadherin has been shown in a number of in vivo studies (Aziz et al., 2009; Chogle, Bu, Wang, & Brown, 2011), however the underlying mechanism still remains partly unsolved. In the dextran sodium sulfate colitis model utilized by Aziz et al. (2009) the anti‐inflammatory and entero‐protective effect of lactadherin was proposed to involve α_v_β_3_ integrin activation and NF‐κB inhibition. This conclusion was however not mirrored in the Bu et al. (2007) study showing no apparent involvement of the integrin‐binding domain of lactadherin. When comparing the published in vivo results to the presented migration data as well as studies by Hanayama et al. (2002), it could be implied that the beneficial bioactive effects of lactadherin in inhibiting inflammatory bowels disease pathology could rely less on induction of enterocyte migration and more on phagocytosis of opportunistic pathogens and apoptotic cells as well as induction of immunosuppressing regulatory T cells (Fava & Danese, 2011; Hanayama et al., 2002; Zhou, Gao, Yang, & Yuan, 2010). A compounding effect from novel evidence of lactadherin as phospholipase inhibitor presents a likely explanation to the anti‐inflammatory and ameliorating effect of lactadherin in inflammatory bowels disease (Nyegaard, Novakovic, Rasmussen, & Gilbert, 2013). Phospholipase inhibition directly correlates with inhibited arachidonic acid release, a crucial eicosanoid precursor utilized in corticosteroid treatment (Cronstein, Kimmel, Levin, & Martiniuk, 1992). As gateway substrate, the inhibition of arachidonic acid release substantially inhibits the synthesis of proinflammatory prostanoids, thromboxane and leukotriene B_4_ important in leukocyte recruitment, extravasion and inflammation (Henderson, 1994; Ricciotti & FitzGerald, 2011). Leukotriene B_4_ inhibition is of particular importance as it highly correlates with inflammatory bowels disease (Hawthorne, Boughton‐Smith, Whittle, & Hawkey, 1992; Sharon & Stenson, 1984).

Hence, increased phagocytosis and inhibition of inflammatory phospholipase activity might largely account for the observed entero‐protective effects of lactadherin in mice and not necessarily relate to enhanced enterocyte migration mediated directly by cell‐lactadherin interactions.

In conclusion, we show that neither recombinant nor purified native lactadherin orthologs from three different species on four different intestinal cell lines induce enterocyte migration. Conversely, 22% inhibition was seen at high lactadherin concentrations (10 μg/ml) and no effect in the serum concentration range of 3–40 ng/ml observed in healthy adults (Cheng, Li, Li, & Wang, 2012; Yamaguchi, Takagi, Miyamae, & Yokota, 2008). The discrepancy of the presently shown results versus previous studies might relate to experimental differences when using silicone inserts versus “scratching,” as ECM and cell damage are likely to play a role in the latter. A more in‐depth discussion of silicone inserts versus scratching/razors can be found in Nyegaard et al. (2016). Despite the uncertainty of the underlying mechanism of the entero‐protective effects of lactadherin, a growing body of literature supports the notion that lactadherin could be used as an entero‐protective agent against colitis, sepsis, LPS‐induced inflammation, ischemia and similar (Ajakaiye, Jacob, Wu, & Yang, 2012; Aziz, Jacob, Matsuda, & Wu, 2011; Cui, Miksa, Wu, & Komura, 2010; Matsuda, Jacob, Wu, & Zhou, 2011; Shah, Wu, Jacob, & Molmenti, 2012). Studies of the efficacy of separate lactadherin domains as well as quantification of direct downstream products in the inflammatory cascades should aid in the elucidation of the immediate mechanisms of this important anti‐inflammatory agent.

## CONFLICT OF INTEREST

The authors declare no competing interests.
